# Supplementary material for: Effectiveness of blended learning in pharmacy education: An experimental study using clinical research modules
Source: PLoS One. 2021 Sep 1;16(9):e0256814. doi: 10.1371/journal.pone.0256814 (PMC8409684; doi:10.1371/journal.pone.0256814)
Supplement: S1 Appendix — (DOCX) [file pone.0256814.s001.docx]

**S1Appendix:**

Motivated strategies for learning questionnaire (MSLQ).

MSLQ contains two sessions, motivation session and a learning strategies session.

Motivation session:

1. Intrinsic goal orientation- student perceive herself to be participating in a task for reasons such as challenge, curiosity, mastery.

2. Extrinsic goal orientation: -Student perceives to be participating in a task for reasons such as grades, rewards, performances, evaluation by others and competition.

3. Task value-evaluation of students on how important or how useful a given task

4. Control of learning belief-students belief that their efforts to learn will result in positive results.

5. Self efficacy-self appraisal of ones ability to master a task

6. Test anxiety-anxiety is negatively correlated to performance .

Learning strategy session:

Rehearsal: Reciting or naming items from a list to be learned. These strategies are assumed to influence the attention and encoding process.

Elaboration: This strategies help students to store information into long term memory and connect new information to prior knowledge.

Organization: Organization strategies help the learner select appropriate information and also construct connections among the information to be learned. Examples of an organizing strategies are clustering, outlining, and selecting the main idea in reading passages.

Critical Thinking: Critical thinking refers to the degree to which students report applying previous knowledge to new situations in order to solve problems, reach decisions, or make critical evaluations with respect to standards of excellence.

Metacognitive Self-

Regulation: There are three general processes that make up metacognitive self-regulatory activities: planning, monitoring, and regulating. Planning activities such as goal setting and task analysis help to activate, or prime, relevant aspects of prior knowledge that make organizing and comprehending the material easier. Monitoring activities include tracking of one's attention as one reads, and self-testing and questioning: these assist the learner in understanding the material and integrating it with prior knowledge. Regulating refers to the fine-tuning and continuous adjustment of one's cognitive activities. Regulating activities are assumed to improve performance by assisting learners in checking and correcting their behaviour as they proceed on a task.

Time and study environment:

Besides self-regulation of cognition, students must be able to manage and regulate their time and their study environments. Time management involves scheduling, planning, and managing one's study time. This includes not only setting aside blocks of time to study, but the effective use of that study time, and setting realistic goals. Time management varies in level, from an evening of studying to weekly and monthly scheduling. Study environment management refers to the setting where the student does his/her class work. Ideally, the learner's study environment should be organized, quiet, and relatively free of visual and auditory distractions.

Effort regulation: Self-regulation also includes students' ability to control their effort and attention in the face of distractions and uninteresting tasks. Effort management is self management, and reflects a commitment to completing one's study goals, even when there are difficulties or distractions. Effort management is important to academic success because it not only signifies goal commitment, but also regulates the continued use of learning strategies.

Peer learning:

Collaborating with one's peers has been found to have positive effects on achievement. Dialogue with peers can help a learner clarify course material and reach insights one may not have attained on one's own.

Help seeking:

Another aspect of the environment that the student must learn to manage is the support of others. This includes both peers and instructors.

Scoring of MSLQ questionnaire:

MSLQ questionnaire contains 81 questions, comprised of 31 questions from motivation session and 50 questions from learning strategy. Students rate in a 7 point response scale ranging from “not at all true of me “to “very true true of me”.

Link for MSLQ questionnaire: <https://files.eric.ed.gov/fulltext/ED338122.pdf>
