# Supplementary material for: Effectiveness of blended learning in pharmacy education: An experimental study using clinical research modules
Source: PLoS One. 2021 Sep 1;16(9):e0256814. doi: 10.1371/journal.pone.0256814 (PMC8409684; doi:10.1371/journal.pone.0256814)
Supplement: S2 Appendix — (DOCX) [file pone.0256814.s002.docx]

**S2appendix:** **Pilot study**

**Objective: To find out the feasibility of study and to explore the students’ experience of using CLINIC-E-LEARNING.**

Educational Activity and Setting: We conducted a workshop on the fundamentals of clinical research for final year pharm D students in the college of pharmaceutical sciences (College name is masked for review). Duration of workshop was four hours. (Complementary lunch was provided to participants). The course was related to the roles and responsibilities of a study coordinator in a clinical trial. The study design was pretest- posttest experimental design followed by focus group discussions. Participants who are interested and obtained a GPA of more than 65% marks in their last university examination were only eligible to participate the study. There were three groups, didactic teaching as control, web-based e-learning and blended learning as experimental groups. Students were randomly allocated to didactic teaching, web-based e-learning and blended learning group by using simple randomization. (Randomization was done by using a software named Graph pad quickcalcs; <https://www.graphpad.com/quickcalcs/randMenu/>) (Suresh, 2011, Graphpad accessed on July 2019) Randomization sequence was generated based on the order registration number.

**Ethics consideration:** This study has been registered in the Kasturba Medical College and Kasturba hospital institutional ethics committee (Ref: 25/2018). Participant information sheet (PIS) was provided to all participants. The principal investigator explained in detail about the study and has taken informed consent form from each and every participant

**Design and Implementation of web based e-learning platform (CLINIC E-LEARNING)**

**Description of the training program:** This training program is designed for students and intended to provide an opportunity to learn about the basics of clinical research and train students on performing clinical trial-related activities (site-based). Learning module comprised of following contents, how to start a clinical trial, site evaluation, site initiation, and Institutional review board procedures. Initial draft content of the learning module was developed by referring text books, articles, regulatory guidelines, and informal discussion with experts. Initial draft content was verified by 5 experts from the relevant area, and based on their suggestion final draft prepared.

**Web based e-learning platform for clinical research (CLINIC E-LEARNING, 2019)**

CLINIC E-LEARNING **(**[**http://clinilearn.in/my/**](http://clinilearn.in/my/)**):**  A new learning management system, CLINIC E-LEARNING ( website link is provided) was designed to create a learning environment, enhance teaching and students’ learning experience. Website is composed of pretest, introduction, 4 videos with full script, summary of all videos as read 1, 2, 3 & 4, case studies, posttest and feedback form. Only eligible participants were allowed to access the learning management system through login id and password.

**Various strategies used for different teaching learning method**

Didactic teaching was conducted by experts in clinical research. The web-based e-learning platform contains the same content of class room teaching. Students were provided with URL, login ID & password. Though all students had knowledge of computer and internet use, we provided a session on how to use the website for e-learning. Students were exposed to both class room teaching and web-based e-learning in blended learning. Same experts delivered the class for didactic teaching and blended learning to avoid bias.

**Outcome measures**

Outcome was measured by validated knowledge questionnaire comprised of multiple choice questions and case study questions. Questions were merely related to subject and validated by 5 experts for its content validity. Questions having item content validity index (ICVI) less than 0.78 removed from the questionnaire. Feedback form administered at the end to know about students’ feedback on the program and satisfaction level.

**Data Analysis**

All quantitative variables were expressed as mean±S.D and qualitative variables were represented in percentage and frequency. Data analyzed by using IBM SPSS version 20. One way Anova used to find the difference of the mean of pretest as well as posttest between learning strategies. Paired t test used to find the significant difference from pre and post test scores in each group. Case study scores were not normally distributed and Kruskal Wallis test used to analyze case study scores between learning strategies. Shapiro-Wilks test conducted to check normality before running statistical test.

**RESULTS**

**Description of Participants:** A total of 22 final year pharm D interns were volunteered to participate in this study from a total strength of 30. Majority of the participants were females (68%), with 21.73% in each group and all are in the age group of 22-24yrs. Baseline characteristics of students are given in Table 1:

Table 1: Baseline characteristics of students

| Sl. No | Variables | Didactic teaching  N (%) | Web-based e learning N (%) | Blended learning  N (%) |
| --- | --- | --- | --- | --- |
| 1 | Number of students | 7(31.81%) | 7(31.81%) | 8(36.36%) |
| 2 | MALE  FEMALE | 2(9.09%)  5(21.73%) | 2(9.09%)  5(21.73%) | 3(13.63%)  5(21.73%) |
| 3 | Age group:22-24 | 7(31.81%) | 7((31.81%) | 8(36.36%) |
| 4 | University GPA>6.5 | 7(31.81%) | 7(31.81%) | 8 (36.36%) |

**Comparison of Subject grade in each pedagogy:** As depicted in table 2, significant differences were observed between pretest and posttest scores in web based (p=0.025) and blended learning strategies (p=0.043). But there was no statistically significant difference in posttest scores between didactic teaching, web-based e -learning and blended learning groups by Anova (p>0.05)(between the group comparison).Also it was found that there is no statistically significant difference in case study scores between learning strategies (p=0.668) but mean score was high in blended learning group.

Table 2: Knowledge score between learning strategies.

| Learning strategy | Pre test  Mean ±S.D | | Post Test  Mean± S.D | | Mean Difference between pre &post | | | P value  (within the group) | |
| --- | --- | --- | --- | --- | --- | --- | --- | --- | --- |
| **Multiple Choice Questions(MCQs)** | | | | | | | | | |
| Didactic teaching | 7±1.15 | | 8.14±1.21 | | 1.14 | | | 0.08 | |
| Web-based e-learning | 5.57±1.39 | | 8.71±1.70 | | 3.14 | | | 0.025 | |
| Blended learning | 6.75±1.98 | | 8.37±1.30 | | 2 | | | 0.043 | |
| P value  (between the group) | 0.217 | | 0.741 | | | 0.301 | |  | |
| **Case study Analysis** | | | | | | | | | |
|  | | Didactic teaching | | Wed-based e-learning | | | Blended | | P value((between the group) |
| Mean ± S.D | | 12.85±2.6 | | 13.57±2.4 | | | 14.37±1.7 | | 0.464 |
| Total Score | | 21.42±2.37 | | 22.28±2.13 | | | 22.75±1.58 | | 0.456 |

**Feedback questions:** Most of the students (90.9%) responded that they prefer blended learning program as it is a mixture of face to face and online tutorials. Of them 72.7% recommended inclusion of more case studies would be helpful to deepen their understanding. Students were satisfied with clarity of the explanation (90.9%, discussion of each module lesson (86%), comprehensive coverage of subject matter and consistency of content with subject objectives and syllabus (90%). A total of 57% of didactic learning students, 85.71 % of web-based e-learning, 100% blended learning students agreed that they are satisfied with workshop.

**FGD results**

Two focus group discussions conducted with 15 students of web based e-learning and blended learning to explore students’ experience. All participants were provided with a participant information sheet and informed consent form was obtained. (PIS and IC for FGD was provided in appendix O). Participants were informed to speak out one at a time so that the recorder can pick up everything that they suggest. It was informed that the entire session would be audio recorded, and confidentiality will be maintained. An independent researcher, who is familiar with FGD methods, conducted the discussion. Each discussion was held in an isolated room to avoid outside hindrance. The choice of language was left to the participants to allow exploration of perception and thoughts. An assistant moderator was present to capture the field notes.

**Data Analysis**: Thematic analysis was used to analyse FGD data. Audio recordings were transcribed to verbatim. The assistant moderator managed the audio recordings to ensure confidentiality and deleted all identifiers. The transcribed data was then read and re-read several times, and the recordings were also listened several times to ensure the transcription's accuracy. Thereafter codes were developed to describe the data. Finally, by combining different codes, subthemes then themes were developed. Any initial codes related to the research question were included in a theme. Results are provided in table 3

**Table 3**: FGD results

| **Themes** | **Subthemes** | **Descriptions** |
| --- | --- | --- |
| Importance of case studies | Students prefer including case studies  Case study explains how problems are solved | “Provide additional case studies”  “Case studies helps to understand real life scenarios” |
| Recommendations | Improvement of content  Technical modification | Case studies should be discussed in class  Case studies are too long. and its take time to understand” So I prefer case study discussion can be conducted in class time.  Video length can be reduced |

Based on suggestion from students involved in pilot study, CLINIC-E-LEARNING-website for clinical research modules was modified. The video length has been shortened and long chapters have been divided into additional parts. Additional case studies included for main study. Focus group discussion result was helpful for designing blended learning for main study. It was decided to utilize class time for case study discussion and practice for important forms in BL, in main study.

**Conclusion of pilot study:** Most of the students (90%) preferred blended learning than traditional and pure e-learning. Though there were no statistically significant differences in knowledge score between teaching methods (due to low sample size), students prefer blended learning. Pilot study result will be helpful for designing and evaluating blended learning programs for large sample sizes
