# Supplementary material for: Effectiveness of blended learning in pharmacy education: An experimental study using clinical research modules
Source: PLoS One. 2021 Sep 1;16(9):e0256814. doi: 10.1371/journal.pone.0256814 (PMC8409684; doi:10.1371/journal.pone.0256814)
Supplement: S3 Appendix — (DOCX) [file pone.0256814.s003.docx]

**S3 Appendix**

Blended learning refers to combination of online and face-to face, whereas web-based-e-learning refers to all education that takes place online, while didactic teaching refers to all learning experiences that take place face-to-face in the classroom

Various strategies used for different teaching learning method (table 4)

| Type of pedagogy | Strategies used: |
| --- | --- |
| Didactic teaching(DT) | Listening to the class and taking notes  Active discussion  Case studies discussion for better understanding  Practical session employing various forms in clinical trial activities |
| Web-based e- learning(WEL) | Students listened e-learning module through website Clinilearn ((http://clinilearn.in/my/): ).  After each video, summary of chapter provided.  Case study discussion  Additional Case studies  Simulated forms for practice.  Important links and files  Assignments  Discussion forum to clarify doubt |
| Blended Learning(BL) | Listening to e- learning modules (Except, additional case studies & simulated forms)  Face to face instruction by experts to reinforce the concept through case studies.  Practical session using various forms used in clinical trial activities  Active discussion |
