# Supplementary material for: Effectiveness of blended learning in pharmacy education: An experimental study using clinical research modules
Source: PLoS One. 2021 Sep 1;16(9):e0256814. doi: 10.1371/journal.pone.0256814 (PMC8409684; doi:10.1371/journal.pone.0256814)
Supplement: S4 Appendix — (DOCX) [file pone.0256814.s004.docx]

**S4 Appendix**

**Web- based e –learning program.**

Clinilearn was designed to provide an opportunity for the student to learn about basics of clinical research. This platform was developed by using moodle platform version 3.2. PHP 7.0x used for coding the contents. Audio-visual slides developed was incorporated by Content Management System. Suitable plugins were used to develop different components *viz* course modules, assignments, feedback etc. Most commonly used plugins listed in the table5.

Table 5: Plugins used:

| **Plugins** | **Component name** | **Description** |
| --- | --- | --- |
| [Portfolio plugins](https://docs.moodle.org/dev/Portfolio_plugins) | Portfolio | To store the content of the module |
| Media Players | Media | To run modules |
| [Legacy assignment types](https://docs.moodle.org/dev/index.php?title=Assignment_types&action=edit&redlink=1) | Assignment | Submission of Assignments |
| [Course formats](https://docs.moodle.org/dev/Course_formats) | Format | Different ways of laying out the activities and blocks in a course |

**Simulated Forms:**

Simulation lab was developed by using the dummy forms in Hypertext Markup Language (HTML). HTML version 5.2 used to develop the dummy forms for the students practice. HTML used different tags and attributes to develop the HTML version of dummy forms. WHATWG used to publish HTML. These published versions incorporated in the website as simulation labs.
