# Supplementary material for: Effectiveness of blended learning in pharmacy education: An experimental study using clinical research modules
Source: PLoS One. 2021 Sep 1;16(9):e0256814. doi: 10.1371/journal.pone.0256814 (PMC8409684; doi:10.1371/journal.pone.0256814)
Supplement: S5 Appendix — (DOCX) [file pone.0256814.s005.docx]

**S5 appendix: QUESTIONNAIRES USED**

1. Knowledge questionnaire
2. MSLQ questionnaire
3. Feedback form

Knowledge questionnaire:

1. KNOWLEDGE QUESTIONNAIRE

Please mark the correct answer:

1. Consequences of Serious adverse events are associated with ?
2. □ Death b) □ Inpatient hospitalization/prolongation of hospitalization c) □ Persistent or significant disability or incapacity, d) □ Congenital anomaly, or birth defect or otherwise life threatening e) □ All of the above
3. If principal investigator comes to know serious adverse event after 24 hrs of occurrence, then?
4. □No need to report b) □ Report as SAE c) □Report as SAE and mention within 24 hrs of PI getting to know of the SAE. D) □ Only mention PI came to know reaction within 24 hrs. so no need to mention as SAE
5. Which form has to submit reporting SAE in schedule Y in India?

□ Appendix X1 b) □ Appendix X11 c) □ Appendix X11 d) □ Appendix X

1. To whom PI has to report SAE?
2. □ Ethics committee, sponsor, DCGI of India b) □ Ethics committee, Head of the department, DCGI of India c) □ Ethics Committee &Sponsor d) □ Ethics committee ,sponsor & CRO
3. Within how many days Sponsor to do analysis and report DCGI on SAE?
4. □ 30 days b) □ 14 days c) □ 45 days d) □ 10 days
5. Who will determine compensation for SAE in India?
6. □ Sponsor b) □ CRO c) □ DCGI d) □ PI
7. If sponsor fails to give the compensation to a trial participant for trial related injury?
8. □ DCGI may suspend or cancel the clinical trial and restrict sponsor/CRO from conducting any further trials b) □ Sponsor can continue this trial c) □ Sponsor may not be able to start any new trial d) □ None of the above
9. Formulae for compensation of SAE causing death
10. □ B*F*R b) □ B*F*S c) □ B*F*H d) □ B*F*T
11. DCGI will intimate sponsor regarding compensation within ------------------------of occurrence of SAE
12. □ 150 days b) □ 100 days c) □ 25 days d) □14 days
13. In case of death or clinical trial related injury sponsor shall pay the compensation within--------- of DCGI order
14. □ 30 days b) □ 10 days c) □5 days d) □14 days
15. Smallest unit of observation captured for a subject in clinical investigation?
16. □ Data element b) □ Data originator c) □ Data element identifier d) □ Electronic prompts
17. Identify the correct statement?
18. Data element identifier helps in identifying data originator, subject to which data element belongs, date & time entered b) Data element identifier helps only in identifying subject to which data element belongs c) Data element identifier won’t be helpful if instruments are sending data into eCRF d) None of the statements are correct
19. Which of the following will give alert to principal investigator about missing data?
20. □ Data element b) □ Data originator c) □ Data element identifier d) □ Electronic prompts
21. Authorized person who transcribe data into eCRF?
22. □ Data element b) □ Data originator c) □ Data element identifier d) □ Electronic prompts
23. All information in original records and certified copies of original records of clinical findings belongs to
24. □ Source document b) □ Data element c) □ Data element identifier d) □ Electronic prompts
25. Which of the following statement is false?
26. □ Electronic source data are data initially recorded in paper format b) □ Source documents means records on which clinical observations are first recorded c) □ If subject use an instrument to transmit data to technology service provider data base, Then source is technology service provider data base d) □ Modifications in CRF can be done only by clinical investigator or delegated person
27. Who should review and electronically sign CRF before submitting to sponsor or regulatory agencies.
28. □ PI b) □ Study coordinator c) □ Patient d) □ CRO
29. Electronic Prompts, Flags, and Data Quality Checks gives alert to PI about ?
30. □ Missing data ,data out of range b) □ ADR occurs other site c) □ Sample size target d) □ Next auditing date
31. Examples of data originator except?
32. □ Sponsor b) □ PI c) □ Study coordinator d) □ Patient
33. Guidelines for informed consent except
34. The Nuremberg Code, 1947 b) The Declaration of Helsinki, 1964 c) The Belmont Report d) ICH GCP e) Schedule H
35. Find out the correct statement
36. Language of the informed consent should be clear, simple, & contains technical terms b) Informed consent is a communication process between the sponsor and the participant c) Life threatening situations, Military operations and Public health emergencies are exception to informed consent d) Tuskegee syphilis study and Nazi research experiments are examples of ethical trials and considered as standard for conducting trials e) Consent is said to be free, if it is affected by coercion
37. Which of the following point emphasizes a meaningful informed consent except?
38. Provide adequate information about the trial participation b) Facilitates the participants understanding of the trial c) Influencing participant to take part in the study d) Provide adequate time to ask questions & to discuss with friends and family members e) Obtain participant’s voluntary agreement to participate
39. COERSION means?
40. act of threatening a person b) Facilitate a person to take part in a study by providing adequate information c) Misleading d) Encouraging e) Providing money to take part in a study
41. LAR is needed when ?
42. Subject is literate b) Subject is illiterate c) Subject is of age below 15 years d) For geriatric patients e) Subject is coming under vulnerable population
43. If both subject & LAR is illiterate, then?
44. Verbal consent &thumb impression from subject and Signature from LAR b) Verbal consent from LAR& thumb impression from LAR c) Verbal consent from impartial witness & thumb impression from LAR d) Verbal consent from impartial witness & thumb impression from subject e) Verbal consent, thumb impression from subject/LAR and signature from impartial witness.
45. What is an assent?
46. Childs agreement to participate in a trial b) Literate participant’s agreement to participate in a trial c) Illiterate participant’s agreement to participate in a trial d) Geriatric participant’s agreement to participate in a trial e) Physically Challenged participant’s agreement to participate in a trial
47. Find out the correct statement?
48. Parents should not sign in assent form b) Written assent must be obtained from children of 12-18 years of age c) Written assent is taken from children of 7-12 years in the presence of their parents/LAR d) Assent is not required for children below 15 years e) All statements are correct
49. Re-consenting is required except?
50. A participant who is unconscious regain consciousness b) A child becomes adult c) If there is a change in study procedure d) Possibility of identity disclosure through data presentation e) Literate participant is participating in a observational trial ( minimal risk )
51. Participant information sheet (PIS) is used to?
52. Document the informed consent procedure b) Information Sheet which provides only the information c) Information sheet provides information about only ADR d) Another term of investigator brochure e) Prepared by investigator to provide participants if they have no time to explain about IC procedure.
53. Identify the correct procedure
54. Advertisement, recruitment, screening, enrollment, randomization b) Advertisement, Screening, recruitment, enrollment, randomization c) Advertisement, randomization, screening, enrollment, recruitment d) None of the above
55. “Participants in a clinical trial do not know which treatment arm they are assigned to” is termed as
56. Blinding b) Masking c) Both a& b d) None of the above
57. Why outcome adjudicator/data analyst is masked in a trial?
58. To avoid the risk that assessors will record more favorable responses b) They may reveal to patients on “trial arms they are” c) None of the above
59. Identify correct statement
60. IRB has responsibility to review advertisement for clinical trial, and have right to approve, modify or reject b) IRB has responsibility to review advertisement, but not supposed to reject c) IRB has no roles in advertisement since it is prior recruitment d) None of the above
61. Advertisement should not use the words excepts?
62. New drug b) New treatment c) Eligibility criteria d) Free treatment
63. Screening of patients is based on inclusion and exclusion criteria and informed consent is not needed. Identify above mentioned statement is true or false?
64. True b) False
65. Last assignment cannot tell anything about next assignment and each assignment is completely unpredictable in?
66. Simple randomization b) Stratified randomization c) Adaptive covariate randomization
67. Identify correct statement?
68. Sample size should be divisible by block size, and block size divisible by number of treatment group. b) Block size should be divisible by sample size, and block size divisible by number of treatment group c) Block size should be divisible by sample size, and block size divisible by number of allocation ratio d) Block size should be divisible by sample size, and number of treatment group divisible by block size e) None of the above
69. Advantage of blocked randomization
70. Next assignment cannot be revealed b) Imbalances can be avoided c) Both a& b d) None of the above
71. If number of covariates increases, chances of imbalances will also be
72. More b) Less c) Not affecting
73. What all are the major documents to be signed before initiating a trial in site?
74. Confidentiality agreement, Investigator undertaking, Clinical trial agreement &Financial disclosure certificate b) Confidentiality agreement, Investigator undertaking, Clinical trial agreement and ADR reporting forms c) Audit certificates, site responsibility logs, and completed, signed & dated CRF d) All of the above
75. Which of the document will be having terms & conditions of indemnification and insurance
76. Confidentiality agreement b) Investigator undertaking c) Clinical trial agreement d) Feasibility questionnaire
77. Find out the correct procedure
78. Confidentiality agreement- feasibility questionnaire-site evaluation-Site initiation b) feasibility questionnaire- Confidentiality agreement-site initiation-site evaluation c) Site initiation-confidentiality agreement-feasibility questionnaire-site evaluation d) Site initiation- feasibility questionnaire-confidentiality agreement- site evaluation
79. How many original copies of CTA has to be made and who all will keep the signed CTA
80. 3;PI,Institution & sponsor b) 4; PI, Institution, sponsor& SMO c) 3;PI,CRO &Institution d) 4;PI,CRO,Institution & SMO
81. Synonym for site selection visit except?
82. Pre selection visit, b) Site evaluation visit , c) Site qualification visit d) Site initiation visit
83. Select the true statement about IEC.
84. Member secretory from outside the institution and chairman from within the institution b) Both member secretory and chairman from within the institution c) Member secretory from within the institution and chairman from outside the institution d) Both member secretory and chairman from institution.
85. Independence & competence are important hallmarks of IEC?
86. True b) False
87. Serious adverse drug reaction to be notified to ethics committee within?
88. 24hrs of awareness b) 1 month c) 7 days d) 45 days
89. Systematic review and meta-analysis will come under
90. Exemption from review b) Expedited review c) Full board review
91. All investigators from different site will participate in
92. Investigator meeting b) Site initiation c) Site evaluation d) Site monitoring.
93. Predesigned printed, optical or electronic document used to record all the protocol required information on each trial subject is termed as
94. Case report form, b) SAE report form c) budget form d) Investigator brochure

Case study questions

1. A 61 years old diabetic female patient involved in in osteoarthritis trial had an increased post prandial blood sugar value which was mild and unrelated to the investigational product. Her PPBS value was 250 mg/dl on previous visit& now it is 500 mg/dl. Hence dosage adjustment & addition of new treatment was required. Is it required to note down since the increase in PPBS not related to osteoarthritis or clinical trial
2. Yes
3. No

2. A Patient was participating in a randomized, double blind, multi-centric, placebo controlled, and phase –II study assessing the safety and efficacy of investigational product in patients with Osteoarthritis. The study duration was one year. During the course of trial he had fever hence he went to local clinic. He was advised antibiotics and tab Dolo 650mg tid for 3 days. However the symptom was not related to the investigational product. What is to be done? Identify correct statement?

1. It needs to be recorded as an adverse event
2. No need to record as adverse event

3. Patient was admitted in our hospital for acute febrile illness. He had septic shock, ARDS, Anemia, Renal failure and hypertension during his admission in ICU for few months. He was better and was shifted to ward. There he developed invasive candidemia and hence enrolled in a Phase III, Double-Blind Randomized Study to Evaluate the Safety and Efficacy of investigational product versus Caspofungin Followed by Voriconazole Regimen for treatment treatment of Candidemia. During course of trial he had gastric irritation and advised Injection Pan. He had on off fever when he was on infusion of Investigational product. The cause of fever was not known and was considered as drug fever and IV drug was shifted to oral antifungal drug. Fever was treated with Paracetomol. In later days of admission during his participation in trial, he had rashes on skin which was diagnosed as keratolysis exfoliate by dermatologist and medicine was given for local application. He had severe leg pain which was diagnosed as mild sensory motor axonal neuropathy and medicine was prescribed by neurologist. He also had left eye irritation for which ophthalmologist prescribed medicines.

Which of the following statement is correct?

1. Gastric irritation, fever, keratolysis exfoliativa, mild sensory motor axonal neuropathy should be recorded as AE.
2. fever, keratolysis exfoliativa, mild sensory motor axonal neuropathy should be recorded as AE but not gastric irritation
3. Gastric irritation, fever, keratolysis exfoliativa, should be recorded as AE but not mild sensory motor axonal neuropathy.
4. All statements are wrong.

4. A subject with COPD enrolls in randomized, placebo controlled, double blind, phase 3 clinical trial evaluating safety and efficacy of new investigational drug. Two weeks after being randomized and started on the study intervention, subject experienced yellowing of skin, pain in right abdomen and abdominal swelling. Subject was admitted in hospital and diagnosed liver failure evidenced from elevated pro thrombin time, aminotransferase level, bilirubin and low platelet count. The known risk profile of the investigational drug does not include any heaptic failure. Evaluation of subject reveals no other cause of liver failure.

Above mentioned episode will be coming under

a) Adverse event

b) Serious adverse event

c) PI has to do causality & severity assessment, then only we can tell it is a AE/SAE

5. Site A was conducting a trial to evaluate the efficacy of a drug for type 2 diabetes mellitus. They were using e CRF for entering data. Here data from glucometer were automatically transmitted to eCRF. Which is source documents from above mentioned scenario?

1. FBS report
2. No source documents
3. eCRF as direct transmission
4. instrument
5. Paper CRF

6. A randomized phase 3 clinical trial, evaluating safety and efficacy of investigational new anti TB drug (along with three anti TB drugs) to a standard therapy. Chest xray, sputum microscopy & other Physical examination has been done during each visit and Physician recorded their progress notes also. Principal investigator entered data into ecrf .Transcription of data from paper to Ecrf was the data capturing method. Do we have to maintain original documents after transcribing into eCRF?

1. Yes, but not mandatory
2. No
3. All original document should be maintained &available to the regulatory agency if requested.
4. PI can store original document for his future reference or publication purpose
5. A subject with chronic asthma participating in randomized, double blind, phase 3 clinical trial evaluating safety and efficacy of new anti-asthmatic drug to standard therapy. Patient reported outcome was the secondary endpoint of the trial to determine whether drug provides any treatment benefit. All subjects were given handheld devices for recording patient reported outcome as to ensure the integrity and accuracy of clinical trial data. These data directly transferred to eCRF. Who will be data originator, If patient is transmitting data directly into ecrf?
6. PI
7. Study coordinator
8. Study sponsor
9. Patient
10. A 2 month old infant admitted in hospital diagnosed with single ventricle congenital heart disorder and doctors asked them to participate in a trial to check the pharmacokinetic and safety profile of digoxin. Digoxin has been approved by the U.S. Food and Drug Administration for the treatment of heart failure in children and adults, more information is needed, particularly in children with single ventricle CHD. Study protocol says study is having more than minimal risk.

PI has to take consent from?

1. Consent from both parents &assent from child
2. Consent from one parent & assent from child
3. Consent from parents only
4. Consent is not necessary as participant is infant
5. None of the above
6. A 16 old obese boy was admitted in hospital due to epileptic seizure. As he met all inclusion criteria, doctor informed about a study on pharmacokinetics of anti-seizure medications (levetiracetum, valproic acid, topiramate, and oxcarbazepine) in obese children to inform dosing guidelines. Parents agreed to participate after the clarification of all doubts they raised. (both are literate and working in govt. sector).

PI has to take consent from?

1. Consent from both parents &assent from child
2. Consent from one parent & assent from child
3. Consent from both parent
4. Consent is not necessary
5. None of the above
6. A multi centric, Phase 3,randomized, double blind study on evaluating effect of new anticancer drug was conducted by ABC pharmaceutical company(sponsor). After one year of trial, they made some changes in study protocol which could affect patients. They have taken IC from all participants before study initiated.

Which of the following statement is correct based on above scenario?

1. Re consenting is required as there is a change in study protocol which could affect patient
2. Continue with the modified protocol, no need of taking IC again
3. Inform the patient about the changes, no need of consent again as they have taken IC 1 year back
4. A 56 year old diabetic patient presents to the emergency department with swelling of feet, ankles and hands, confusion, and loss of appetite. Provisional diagnosis of the patient was diabetic nephropathy. A clinical trial of diabetic nephropathy was going on in the same hospital that time. So he was ready for participating & asked to consult a receptionist who follows a script to determine basic eligibility for the specific study. They found that he is not eligible for the study as he is not met all inclusion criteria.

Does IRB has any roles here as patient is excluded from the study?

1. IRB does not have any roles as patient is not recruited for study
2. The IRB should assure the procedures followed adequately protect the rights and welfare of the prospective subjects
3. A multinational pharmaceutical company made a statement like all participating subjects will get free medical treatment for the entire duration of clinical trial in an advertisement for recruitment of subjects for a open labeled uncontrolled clinical study evaluating the effectiveness of new oral anti psoriasis agent.

Is there any ethical issue in above mentioned advertisement?

1. Yes, it is a uncontrolled trial
2. Yes, they have “mentioned free medical treatment”
3. No. As it is an advertisement to recruit participants, IRB does not even role.
4. Research study to evaluate new drug to improve quality of life in rheumatoid arthritis along with exercises was conducted in a tertiary care hospital. End point was based on disease process measures, joint count, erythrocyte sedimentation rate, C-reactive protein and semi-structured interview with patients on their quality of life after 3 months. If it is a triple blinded study, who all were masked from study (Please note that PI appointed study coordinator for data entry and conducting interview).
5. PI, Patient & Patient representative
6. PI, Patient& Pharmacist
7. PI, Patient & Study Coordinator
8. None of the above
9. Which of the following randomization will be more suitable for a study to evaluate the effect of yoga on memory in a tertiary care hospital. Inclusion criteria is people on all age group, both genders and with no comorbidities.
10. Simple randomization
11. Stratified randomization
12. Non probability convenience sampling
13. Snowball sampling
14. & 16. Mr. Kim was working as a study coordinator in a tertiary care hospital. Director of site monitoring organization (SMO) received a mail recently from ABC pharmaceuticals asking their interest to participate in a phase 3 trials. Director of SMO found potential investigator from hospital and appointed Mr. Kim for further follow ups.

Principal investigator received confidentiality agreement from sponsor, he completed and signed

Sponsor sent feasibility questionnaire to principal investigator. Does Kim have any responsibilities at this stage?

1. Help investigator to fill the feasibility questionnaire in terms of whether thy will be getting adequate patient population, do they need additional collaboration, do they need additional space or equipment…
2. Filling feasibility questionnaire is mere responsibility of Principal investigator. Mr.Kim no need to do anything

After submitting the FQ, site received a mail intimating site evaluation visit,Ms. Susan, Clinical research associate (CRA, representative of clinical research organization) came for site evaluation visit. Does kim have any responsibility now?

1. Susan will meet principal investigator only. Kim does not have any responsibility at this stage
2. Susan will meet principal investigator and she will explain in detail about protocol. Mr.Kim has to be actively participated in discussion. Then Ms. Susan will conduct department visit with Mr.Kim to ensure site has adequate facilities to perform protocol.
3. ABC pharmaceuticals approached Dr. John, nephrologist for conducting phase 3 trials. Head of SMO appointed Mr. Kim for assisting Dr. John. Dr John signed confidential agreement and completed feasibility questionnaire with Mr. Kim. Ms. Susan, CRA conducted site evaluation visit and site received a mail regarding site selection. After site selection, does Dr. John need to do anything before recruitment of patients?
4. Dr. John has to only submit documents for ethics committee clearance.
5. Dr john has to submit financial disclosure, principal investigator undertaking, signed CTA and ethics committee clearance to sponsor .If there are any changes of approved protocol, Dr. John has to intimate ethics committee too.

2. MSLQ questionnaire: <https://files.eric.ed.gov/fulltext/ED338122.pdf>. MSLQ questionnaire is available in public domain.

**3. Feedback form**

**Feedback form**

Kindly place a tick (√) mark in the chosen response column and remarks in the remark column of the scale.

| Excellent | Good | Fair | Poor |
| --- | --- | --- | --- |
| 4 | 3 | 2 | 1 |

| Sl. No: | Statement | 4 | 3 | 2 | 1 |
| --- | --- | --- | --- | --- | --- |
| 1 | Design of the modules |  |  |  |  |
| 2 | User-friendliness |  |  |  |  |
| 3 | Explanation of purpose, objectives, and grading procedures |  |  |  |  |
| 4 | Clarity of explanation and discussion of the module lessons. |  |  |  |  |
| 5 | Consistency of content with subject objectives and syllabus |  |  |  |  |
| 6 | Comprehensive coverage of subject matter |  |  |  |  |
| 7 | Relevance of hyperlinks (if any) |  |  |  |  |
| 8 | Graded quizzes |  |  |  |  |

OVERALL SATISFACTION OF STUDENTS

Instruction: Kindly place a tick (√) mark in the chosen response column and remarks in the remark column of the scale

Do you agree the following Statements?

| Sl.No: | Statements | Strongly agree  (5) | Agree  (4) | Neutral  (3) | Disagree  (2) | Strongly Disagree  (1) |
| --- | --- | --- | --- | --- | --- | --- |
| 9 | Attained subject objectives |  |  |  |  |  |
| 10 | Accomplished various learning activities |  |  |  |  |  |

1. Do you agree that you are satisfied with this program
2. Strongly agree
3. Agree
4. Neutral
5. Disagree
6. Strongly Disagree
7. Which learning strategy do you prefer?
8. Blended learning
9. Web-based e-learning Program
10. Class room teaching

**FEEDBACK FORM FOR DIDACTIC TEACHING**

Kindly place a tick (√) mark in the chosen response column and remarks in the remark column of the scale.

| Excellent | Good | Fair | Poor |
| --- | --- | --- | --- |
| 4 | 3 | 2 | 1 |

| Sl. No: | Statement | 4 | 3 | 2 | 1 |
| --- | --- | --- | --- | --- | --- |
| 1 | Explanation of purpose, objectives, and grading procedures |  |  |  |  |
| 2 | Clarity of explanation and discussion of the module lessons. |  |  |  |  |
| 3 | Consistency of content with subject objectives and syllabus |  |  |  |  |
| 4 | Comprehensive coverage of subject matter |  |  |  |  |

5. Which learning strategy do you prefer?

1. Blended learning
2. Web-based e-learning Program
3. Class room teaching

6. Do you agree that you are satisfied with this program

1. Strongly agree
2. Agree
3. Neutral
4. Disagree
5. Strongly Disagree
