# Supplementary material for: Effectiveness of blended learning in pharmacy education: An experimental study using clinical research modules
Source: PLoS One. 2021 Sep 1;16(9):e0256814. doi: 10.1371/journal.pone.0256814 (PMC8409684; doi:10.1371/journal.pone.0256814)
Supplement: Attachment — Submitted filename: Rebuttal letter.docx [file pone.0256814.s008.docx]

REBUTTAL LETTER (ARTICLE REFERENCE NUMBER: PONE-D-20-24489R1).

Dear sir/madam,

Herewith attach comments in revision 1 and rejection mail. There were 4 reviewers in first stage and 3 in second stage. Summary of comments given by reviewers in Revision 1 and rejection mail

|  |  | Comments in Revision 1 |  | Comments in Rejection mail |
| --- | --- | --- | --- | --- |
| Is the manuscript technically sound, and do the data support the conclusions? | Reviewer 1 | Yes | Reviewer 1(M. Chandra Sekar,) | yes |
|  | Reviewer 2(APARNA YERRAMILLI) | Yes | Reviewer 2(APARNA YERRAMILLI)) | yes |
|  | Reviewer 3 | Yes | Reviewer 3 | partly |
|  | Reviewer 4 | yes |  |  |
| Has the statistical analysis been performed appropriately and rigorously? | Reviewer 1 | Yes | Reviewer 1 | Don’t know |
|  | Reviewer 2 | Yes | Reviewer 2 | yes |
|  | Reviewer 3 | Yes | Reviewer 3 | yes |
|  | Reviewer 4 | yes |  |  |
| Have the authors made all data underlying the findings in their manuscript fully available? | Reviewer 1 | Yes | Reviewer 1 | yes |
|  | Reviewer 2 | Yes | Reviewer 2 | yes |
|  | Reviewer 3 | Yes | Reviewer 3 | yes |
|  | Reviewer 4 | yes |  |  |
| Is the manuscript presented in an intelligible fashion and written in standard English? | Reviewer 1 | Yes | Reviewer 1 | yes |
|  | Reviewer 2 | NO | Reviewer 2 | yes |
|  | Reviewer 3 | Yes | Reviewer 3 | yes |
|  | Reviewer 4 | yes |  |  |
| If the authors have adequately addressed your comments raised in a previous round of review and you feel that this manuscript is now acceptable for publication, you may indicate that here to bypass the “Comments to the Author” section, enter your conflict of interest statement in the “Confidential to Editor” section, and submit your "Accept" recommendation | Reviewer 1 |  | Reviewer 1 | All comments have been addressed |
|  | Reviewer 2 |  | Reviewer 2 | All comments have been addressed |
|  | Reviewer 3 |  | Reviewer 3 | No response |
|  | Reviewer 4 |  |  |  |
|  |  |  |  |  |

Reviewer comments:

| Reviewer | Comments with Revision 1 | Reviewer | Comments in rejection |
| --- | --- | --- | --- |
| Reviewer 1 | I enjoyed reading this manuscript. This research becomes more timely as a result of COVID's impact on higher education around the world. Study was carried out with students from multiple colleges and with both control and interventions done at the same time. | Reviewer 1 | In line 307, author use the word "against". I believe "contrast" is more appropriate. In line 349 - "Covid awake" not sure what it means. Did they meant to say - in wake of Covide? Limitations of the studies has been well articulated. |
| Reviewer 2 | The language used needs to have more clarity. The tense needs to be uniform throughout. It is not clear if the duration of the learning program was the same for all the three arms. The results and the changes to the modules resulting from the pilot study observations is not presented. The GPA conversion from the % is not clear (>6.5 ??) A modified MSLQ questionnaire with 44 questions was used in reference 11. In the study 81 questions were used which is lengthy and time consuming. The authors could elaborate why a lengthy quesionnaire was used. | Reviewer 2 | The study has been conducted well and the findings are useful to the present scenario. However improvements can be made to the writing style by taking the assistance of a native English speaker. |
| Reviewer 3 | English language usage for the U.S. needs to be addressed. The authors have a done a good job of explaining the various learning methodologies and their distinctions. This study seems to be the first of its kind in developing countries and as such is a good contribution to the overall education literature. | Reviewer 3 | This paper presents comparison of two types of learning methods. The paper is an educational research paper and may be better suited in an educational journal such as Currents in Pharmacy Teaching and Learning or American Journal of Pharmaceutical Education. The biggest flaw in the argument of this paper is the proposition that teaching clinical research to students would result in their employability as researchers with Contract Research Organizations. The sample sizes were drawn from Southern India whereas most CROs are based in Northern India where cultural and other differences should be taken into account. Skillset building is a longitudinal process and while it is noteworthy that the authors compared and contrasted these methods, the sample size (n=241), and cross-sectional nature of this study are insufficient to draw adequate conclusions about any methodology. The paper needs copyediting to ensure that it meets grammatical standards for any U.S. journal. |
| Reviewer 4 | update tables as per journal style. Paper is well written,. BL gr-pls add more details in methodology as it seems similar to others 2 groups. COVID is mentioned in discussion, but not in intro,... similar clinical research module is highlighted in intro but not in discussion. |  |  |

Reviewer 1 & 2 have written all comments in revision 1 have been addressed. Comments of reviewer 1 & reviewer 2 in rejection mail is only related to improvements in writing style and grammatical error, which is addressed in the revised manuscript.

**Point by point response to reviewer 3 in rejection mail.**

| This paper presents comparison of two types of learning methods | **Paper compares Three(3) type of learning strategies** |
| --- | --- |
| The paper is an educational research paper and may be better suited in an educational journal such as Currents in Pharmacy Teaching and Learning or American Journal of Pharmaceutical Education | Plos one has published educational articles before also . We know Plos one has its own Education Research Collection. Thatswhy we chose this journal. |
| The biggest flaw in the argument of this paper is the proposition that teaching clinical research to students would result in their employability as researchers with Contract Research Organizations | **This articles deals with comparison of various learning strategies (Didactic, web-based,& blended) in terms of knowledge score , SRL use and students satisfaction. We have not written anywhere in this manuscript as** teaching clinical research to students would result in their employability as researchers with Contract Research Organizations. We have mentioned in discussion, training in these areas would be useful for students to bag a job. Not mentioned any kind of job/clinical research organization. Also we have added this statement in view of reviewer 4 in first revision. In first revision, reviewer 4 had asked why clinical research module is not mentioned in discussion. We want to justify why we chose clinical research as a subject to deliver learning strategies.  Paper deals with comparison of effectiveness of various learning strategies in terms of knowledge score, SRL use and students satisfaction. Also clearly written topics are related to site based clinical trial trial activities. We have used case study practical aspects to teach students. (See description of training program and contents) |
| The sample sizes were drawn from Southern India whereas most CROs are based in Northern India where cultural and other differences should be taken into account | **Why mentioning about CROs ? What’s the point of telling most CROs are based in Northern India. We have not mentioned anything about CRO in this article. After getting a job in CRO, We do not check skills of students who have completed this training program. It’s an educational research and our target population is final year Pharm D students. Our aim is to evaluate the effectiveness of blended learning in pharmacy education. So we chose clinical research as a subject to deliver this.**  **It is cluster randomized trial with 12 colleges from 3 geographical area. And only interested students participated in this study. This has been done to avoid contamination bias. Its written clearly in methodology how we have selected colleges and students.** |
| Skillset building is a longitudinal process and while it is noteworthy that the authors compared and contrasted these methods, the sample size (n=241), and cross-sectional nature of this study are insufficient to draw adequate conclusions about any methodology | **We know skill set building is longitudinal process. Please note that we have analysed knowledge score (MCQs+Case study questions) after completing various learning strategies.**  **Main research gap of previous articles to evaluate effectiveness of blended learning in pharmacy education ( in terms of knowledge score) was, they all have used previous year students(historical cohort) score as control. Also sample size was less. In order to bridge that gap, we conducted a prospective interventional study with minimum bias. As far as we know, there is no similar kind of research has been conducted from developing countries (See reviewer 3 comments in revision 1). Most of the blended learning article in pharmacy education are from developed countries and majority of them used historical controls.**  **Also major drawback of most of the educational studies are, they are not assessing long term effect. We have clearly described this in limitation and future research lines. (See Reviewer 1 comment in rejection mail).** |
| The paper needs copyediting to ensure that it meets grammatical standards for any U.S. journal | **We agree this and happy to do language correction. We are ready to give professional service (All reviewers agreed, manuscript is written in standard English. Since we are not native speakers, we know mistakes will happen. we are ready to give professional service, correction, if article accepted)** |
|  |  |
