# Supplementary material for: Effectiveness of blended learning in pharmacy education: An experimental study using clinical research modules
Source: PLoS One. 2021 Sep 1;16(9):e0256814. doi: 10.1371/journal.pone.0256814 (PMC8409684; doi:10.1371/journal.pone.0256814)
Supplement: Attachment — Submitted filename: PONE-D-21-09098_reviewer.docx [file pone.0256814.s009.docx]

| **PAGE & LINE NO** | **COMMENTS** |
| --- | --- |
| **Title page** |  |
| Page 7 line no 14 | Kindly provide author professional background specification. |
| **Abstract** |  |
| Page 8 line no 37 | It is advised to include the location name where the study has been conducted. |
| **Introduction** |  |
| Page 10, line no 72 | Kindly put reference no. before full stop. |
| **Methods** |  |
| Page 12, line no 125 | Kindly rewrite this sentence. |
| Page 13, line no 149 - 150 | Kindly reform this sentence as meaning is not clear. |
| Page 14, line no 156-157 | Kindly write this sentence in more appropriate way, meaning is contradicted. |
| Page 14, line no 170 | Kindly check for sentence “Same slides of website” meaning is not clear. Rewrite it in more appropriate way |
| Page 15, line no 179 | Kindly remove one bracket. |
| **Results** |  |
| Page 17, line no 229 | Kindly revised the caption for table no 3. |
| Page 20 | Kindly write “Web Based” instead of “We based” in table no 5 |
| **Discussion** |  |
| Page 21, line no 270- 272 | Suggested to remove this sentence as it is not linked with context of manuscript. |
| **Conclusion and future research lines** |  |
| Page 24, line no 350 | Kindly give space in between “COVID 19 & It” |
| **Acknowledgement** |  |
| Page 25, line no 360- 361 | Kindly check for grammatical error. |
| **References** |  |
| Page 25, line no 365- 367 | Kindly rewrite reference in accordance with prescribed guidelines. |
| Page 25, line no 371- 373 | Kindly rewrite reference in accordance with prescribed guidelines. |
| **Summary** | - Location of population must be specified clearly. - Check for grammatical errors and reform sentence with clear elucidation. - While writing references guidelines provided by journal should be followed. - After reviewing this manuscript, it seems that, it needs some corrections before acceptance. |
