# Supplementary material for: Effectiveness of blended learning in pharmacy education: An experimental study using clinical research modules
Source: PLoS One. 2021 Sep 1;16(9):e0256814. doi: 10.1371/journal.pone.0256814 (PMC8409684; doi:10.1371/journal.pone.0256814)
Supplement: Attachment — Submitted filename: PONE-D-21-09098_reviewer.docx [file pone.0256814.s011.docx]

| **PAGE & LINE NO** | **COMMENTS** |
| --- | --- |
| **Methods** |  |
| Page 6, line no 121 | Kindly reframe this sentence with clear elucidation. |
| Page 6, line no 123- 124 | Kindly check for typographical error, either write summary or details of the study. |
| Page 7, line no 147 | Kindly correct spelling of “password”. |
| Page 7, line no 147- 151 | Kindly reform this sentence with clear elucidation. |
| **Results** |  |
| Page 11, line no 233 | Kindly correct Experimental Group 1(Web-based eLearning) Mean Difference Value. Value must be written down to the same decimal place as rest of the values in table. |
| Page 12, line no 247 | Kindly revised the caption for table no 3. |
| **Summary** | - Check for grammatical errors and reform sentence with clear elucidation. - Caption of the tables should be revised and value must be written down to the same decimal place |

After reviewing this manuscript, it seems that abstract write up is up to the mark but some typographical and grammatical error must be corrected. Author have adequately addressed comments raised in a previous round of review. Flow and clarity of manuscript is average and needs minor corrections before acceptance.

1. Appropriateness of the Subject matter – average

2. Methods used - adequate

3 Information Contained - Valuable

4 Clarity of Presentation – Average

5 Statistical analysis - adequate

6 Conclusions – supported objective

7 Language – Average

8 Overall Quality - Average

9 If acceptable, is it possible to reduce text? - Yes

10 Ethical Clearance – required
