# Supplementary material for: Effectiveness of blended learning in pharmacy education: An experimental study using clinical research modules
Source: PLoS One. 2021 Sep 1;16(9):e0256814. doi: 10.1371/journal.pone.0256814 (PMC8409684; doi:10.1371/journal.pone.0256814)
Supplement: Attachment — Submitted filename: REviewer Comments.docx [file pone.0256814.s012.docx]

| **PAGE & LINE NO** | **COMMENTS** |  |
| --- | --- | --- |
| **Methods** |  |  |
| Page 6, line no 121 | Kindly reframe this sentence with clear elucidation. | Corrected |
| Page 6, line no 123- 124 | Kindly check for typographical error, either write summary or details of the study. | Corrected |
| Page 7, line no 147 | Kindly correct spelling of “password”. | Corrected |
| Page 7, line no 147- 151 | Kindly reform this sentence with clear elucidation. | Corrected |
| **Results** |  |  |
| Page 11, line no 233 | Kindly correct Experimental Group 1(Web-based eLearning) Mean Difference Value. Value must be written down to the same decimal place as rest of the values in table. | Corrected |
| Page 12, line no 247 | Kindly revised the caption for table no 3. | Corrected |
| **Summary** | - Check for grammatical errors and reform sentence with clear elucidation. - Caption of the tables should be revised and value must be written down to the same decimal place | -Grammatical errors corrected  -Sentence reframed with clear elucidation  -Table caption modified  -Values checked to the same decimal place |
